# A systematic review of the cost of data collection for performance monitoring in hospitals

**DOI:** 10.1186/s13643-015-0013-7

**Published:** 2015-04-01

**Authors:** Cheryl Jones, Brenda Gannon, Abel Wakai, Ronan O’Sullivan

**Affiliations:** Centre for Health Economics, The University of Manchester, Oxford Rd, Manchester, M13 9PL UK; Department of Emergency Medicine, Beaumont Hospital, Beaumont Rd, Dublin, Ireland; Emergency Care Research Unit (ECRU), Division of Population Health Sciences, Royal College of Surgeons in Ireland (RCSI), 123 Saint Stephen’s Green, Dublin, Ireland; Paediatric Emergency Research Unit (PERU), National Children’s Research Centre, Gate 5, Our Lady’s Children’s Hospital, Dublin, Ireland; School of Medicine, University College Cork, Room 2.59, Brookfield Health Sciences Complex, College Road, Cork, Ireland

**Keywords:** Quality indicators, Healthcare, Quality improvement, Hospitals

## Abstract

**Background:**

Key performance indicators (KPIs) are used to identify where organisational performance is meeting desired standards and where performance requires improvement. Valid and reliable KPIs depend on the availability of high-quality data, specifically the relevant minimum data set ((MDS) the core data identified as the minimum required to measure performance for a KPI) elements. However, the feasibility of collecting the relevant MDS elements is always a limitation of performance monitoring using KPIs. Preferably, data should be integrated into service delivery, and, where additional data are required that are not currently collected as part of routine service delivery, there should be an economic evaluation to determine the cost of data collection. The aim of this systematic review was to synthesise the evidence base concerning the costs of data collection in hospitals for performance monitoring using KPI, and to identify hospital data collection systems that have proven to be cost minimising.

**Methods:**

We searched MEDLINE (1946 to May week 4 2014), Embase (1974 to May week 2 2014), and CINAHL (1937 to date). The database searches were supplemented by searching for grey literature through the OpenGrey database. Data was extracted, tabulated, and summarised as part of a narrative synthesis.

**Results:**

The searches yielded a total of 1,135 publications. After assessing each identified study against specific inclusion exclusion criteria only eight studies were deemed as relevant for this review. The studies attempt to evaluate different types of data collection interventions including the installation of information communication technology (ICT), improvements to current ICT systems, and how different analysis techniques may be used to monitor performance. The evaluation methods used to measure the costs and benefits of data collection interventions are inconsistent across the identified literature. Overall, the results weakly indicate that collection of hospital data and improvements in data recording can be cost-saving.

**Conclusions:**

Given the limitations of this systematic review, it is difficult to conclude whether improvements in data collection systems can save money, increase quality of care, and assist performance monitoring of hospitals. With that said, the results are positive and suggest that data collection improvements may lead to cost savings and aid quality of care.

**Systematic review registration:**

PROSPERO CRD42014007450.

## Background

Key performance indicators (KPIs) are used to monitor performance in key areas of a service. These KPIs are implemented at hospital level and are used to identify where organisational performance is meeting desired standards, and where performance requires improvement. They enable the public, service users, and healthcare providers alike to have reliable information on current and desired standards in healthcare services [1]. However, the feasibility of collecting the relevant minimum data set ((MDS) the core data identified as the minimum required to measure performance for a KPI) elements is always a limitation of performance monitoring using KPIs. For example, in a pilot feasibility analysis of four potential Emergency Department (ED) KPIs, approximately half of the relevant MDS items were missing in the patient records [2].

The reporting burden of capturing the relevant MDS elements should not outweigh the value of information when using KPIs for performance monitoring [1]. Preferably, data should be integrated into service delivery, and, where additional data are required that are not currently part of service delivery, there should be an economic evaluation to determine the cost of collecting all the relevant MDS elements [1]. KPIs are often used in the measurement of costs (or benefits) of data collection, and there is, therefore, a need for a systematic review which synthesises and coheres the evidence base regarding economic analyses of hospital data collection for performance monitoring purposes.

The aim of this systematic review was to synthesise the evidence base concerning the costs of hospital data collection for performance monitoring using KPIs and to identify hospital data collection systems that have proven to be cost-minimising. The review also aimed to identify published studies that addressed the benefits of hospital data collection for performance monitoring and to summarise the methods used to evaluate hospital data collection for performance monitoring purposes.

## Methods

### Research objectives

A systematic review was carried out to identify all published economic analysis and costing studies regarding hospital data collection for performance monitoring. The review was conducted using the methods detailed in the published protocol of the review [3].

### Electronic search

The electronic databases used to search for relevant publications included MEDLINE (1946 to May week 4 2014), Embase (1974 to May week 2 2014), and CINAHL (1937 to date) via the Ovid interface. The electronic search strategies were created specifically for each database using relevant index and free text terms. The full search strategy used can be found in the Appendix. We also limited the search to English language publications.

#### Searching other resources

Additional efforts were made to identify eligible studies by cross-referencing from the reference lists of major publications on the subject and published government reports [3]. We also made additional efforts to identify potential studies relevant to the topic from a ‘grey literature’ (theses, internal reports, non-peer reviewed journals) database (OpenGrey - system for information on grey literature in Europe).

### Inclusion criteria

The inclusion criteria were specified based on the type of study conducted, the population involved with data collection, and the intervention that was adopted to record and collect data.

The studies that were included in the review were economic evaluations and cost or feasibility studies that examined hospital level data collection for performance monitoring purposes using KPIs. The definition of KPIs refers to clinical and quality-of-care indicators such as time to treatment and time to initial assessment. Both types of KPI were accepted for inclusion into the review.

There were two broad categories of participants that were involved with data collection, including health professionals, such as doctors and nurses, and non-clinical staff, such as administrators and managers. All studies that used patient reported data were excluded for the purposes of this review.

All types of interventions (as defined by the authors) that collect and record data for the purpose of monitoring performance were included in the review.

### Study selection

Two researchers (BG and CJ) independently screened the titles and abstracts of the identified studies and assessed the inclusion of studies using specific inclusion and exclusion criteria (see Table 1) [3]. All studies that analysed costs and benefits/effects of data collection for performance monitoring were included in the review. One researcher (CJ) read the papers in full and re-assessed them for inclusion. If there were discrepancies with study selection decisions, and the two investigators who independently screened the potentially eligible studies could not reach a consensus, we planned to resolve the disagreement through discussion and consultation with a third investigator (AW) [3].

**Table 1:** **Inclusion of studies using specific inclusion and exclusion criteria**

**Inclusion criteria**	**Outcome**	
	**Yes**	**No**
Economic evaluation or cost/feasibility study		
Data collection or quality/clinical indicator study		
Hospital/secondary care context		
English or English translation		
Assessment		

Further instructions for inclusion: for the purpose of this review, the definition of KPI will include any variable or a synonym of an indicator used to measure key areas of a service for performance monitoring purposes. Therefore, studies examining quality-of-care indicators and clinical indicators will be screened for inclusion.

### Data extraction

The purpose of this review was to report the hospital costs and benefits associated with data collection. Information concerning the costs and benefits of data collection were extracted regardless of whether any formal KPIs were implemented at the hospital in the study. One author extracted the data using a tailored data collection form as previously described [3]. The data extraction form was based on a checklist developed by the Consolidated Health Economic Evaluation Reporting Standards (CHEERS) [4]. The data extracted incorporated (i) author, year of publication, and country; (ii) intervention; (iii) study population and setting; (iv) methodology and study design; (v) resources; (vi) costs; and (vii) results. The results were then summarised as part of a narrative synthesis.

### Quality assessment

All studies were assessed for their reporting quality. The quality assessment (QA) was based on the CHEERS checklist [4] and was incorporated into the data extraction tables. Because this systematic review was not reviewing typical medical interventions, such as improved screening strategies or new drugs, it was decided that it was unnecessary to assess each study using the full CHEERS checklist. Therefore, with careful consideration, aspects of the CHEERS checklist were included as part of the QA. Table 2 lists the criteria chosen to assess the studies. The results of the QA were tabulated as part of the data extraction process. A description regarding the quality of reporting is presented below.

**Table 2:** **Quality assessment criteria**

**Quality assessment criteria**	**Good**	**Poor**
Intervention and comparator		
Objective and study type		
Setting, population, perspective		
Costs		
Benefits		
Results and conclusions		

## Results

### Results of the search

**Figure 1:**
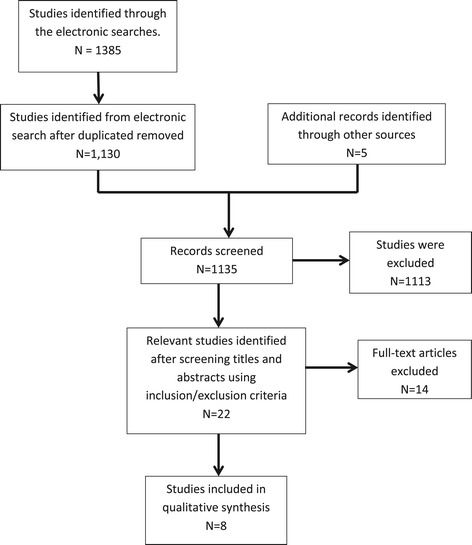
**Study PRISMA flow diagram.**

The electronic search yielded a total of 1,130 publications, once duplicates were removed, and five papers were identified through other sources. After reviewing the titles and abstracts of the publications, we retrieved 22 full-text versions of publications for possible inclusion into the review. Once the full-text versions were examined, we excluded a further 14 publications. The selection process resulted in the identification of eight relevant studies and is summarised using a Preferred Reporting Items for Systematic Reviews and Meta-Analyses (PRISMA) [5] (flow diagram presented in Figure 1) (Table 3).

**Table 3:** **Data extraction and quality assessment results**

**Study**	**Intervention and control**	**Objective and type of study**	**Setting, population, and perspective**	**Costs**	**Benefits**	**Results and conclusions**
Holloway *et al*. [6]	Intervention: computerised electronic records systems, PAS-MAP	Compare differences in completeness, timeliness, operability, and cost	Setting: 214-bed general hospital was studied	Differences in costs of PAS-MAP and manual system including: data abstraction costs, subscriptions, and summary preparation time	Completeness	Costs: the manual system would cost $2,593 more per year than the PAS
	Comparator: manual system, hand written records	Type of study: cost analysis	Three departments: general practice, medicine, and surgery		Timeliness	Manual system more complete, as timely, and more likely to prevent human error
			Population: physicians, medical admin staff		Operability	
			Perspective: not stated			
Klimt *et al*. [7]	Intervention: Dictaphone for transcribing records	Compare the costs and benefits of transcribing technology against the manual system	Setting: Emergency Department	Cost of average length of record, true transcriber cost (including salary cost, bonus), true productivity of transcriber. Equipment costs are reported	Completeness	Incremental cost of typing an emergency record is $1.03
	Comparator: manual system	Type of study: cost minimisation analysis	Population: physicians and surgeons		Timeliness	Transcribed medical records more complete, less timely, and more accurate
			Perspective: not stated		Operability	
					Accuracy of billings	
Tierney *et al*. [8] USA (Indiana)	Intervention: computerised inpatient orders	To assess the effects on healthcare resource utilisation of a network of microcomputer workstations for writing all inpatient orders	Setting: inpatient internal medicine service hospital	Total costs which include: bed costs, test costs, drug costs, and other costs. Equipment and installation costs are reported	Total charges	Total costs with workstations: $594 less (10.5% lower bed costs, 12.4% lower tests costs, 15.1% lower drug costs)
	Comparator: normal practice	Type of study: cost- consequence analysis	Population: inpatients, house officers, medical students, and faculty internists		Hospital length of stay	Hospital length of stay declined by 0.89 days
			Perspective: not stated		Benefits speculated	
Philp *et al*. [9] UK	Intervention: Information system for monitoring impact of acute hospital care on health status	Develop a patient information system which could be used to evaluate the effectiveness of multidisciplinary hospital care	Setting: Hospital	Staff time, printing, statistical analysis, computing equipment and system administration.	Nurse perspective:	Total annual cost per ward £6,455 to incorporate follow-up assessments
	Comparator: normal practice	Type of study: cost analysis	Population: physicians, nurses, and junior physicians		Decision-making	Undecided if decision-making, teamwork, professional care, and performance was improved
			Perspective: not stated		Teamwork	Benefits for patient care can only be inferred, not proven
					Professional care	
					Performance	
Willems *et al*. [10] Belgium	Intervention: follow-up programme that informs physicians of their compliance and outlines the financial consequences of using prophylactic antibiotics	Evaluate the follow-up programme	Setting: post-operative surgery and obstetrics care	Cost of antibiotic use	Benefits speculated	Total cost of antibiotic use reduced by 50%
	Comparator: previous practice	Type of study: cost analysis	Population: physicians			An average loss of €92,353 pre-intervention became profit average of €27,269 post-intervention
			Perspective: hospital			
Barnes *et al*. [13] USA (Ohio)	Standardisation of coding	Compare volumes, length of stay, and billings volume before and after implementation intervention	Setting: Trauma Care and Surgery Department	Costs are not reported	Hospital length of stay	Increase of $270.46 (394%) on average SHC revenue per trauma service admission
	Comparator: no standardisation	Type of study: not clear	Population: physicians		Completeness	More consistent and complete documentation of patient care.
			Perspective: not stated		Accuracy of billings	
Encinosa and Bae [11]	Intervention: Basic Electronic Medical Records (EMRs)	Assess whether EMRs prevent hospital-acquired conditions (HACs), death, readmissions, and high spending	Setting: inpatient and outpatient departments	Average cost of patient safety event	Probability of death and readmission	Excess spending on patient safety events declines by $4,849 or 16% due to basic EMRs
	Comparator: no basic EMRs	Type of study: cost effectiveness analysis	Population: physicians and patients	IT capital and operation costs		EMRs had no impact on the probability of a patient safety event occurring
			Perspective: not stated			EMRs reduce the probability of readmission once a patient safety event occurs
Encinosa and Bae *et al*. [12]	Intervention: quality indicator based on five core MU elements	Compare the costs and effects of using up to five elements within a quality indicator	Setting: inpatient departments	All hospital costs were included except physician and laboratory costs (no justification as to why these were left out and no table to describe what costs were included)	Averted adverse drug event	Estimated costs savings at $4,790 per averted adverse drug event
	Comparator: use of 0 to 5 elements	Type of study: cost effectiveness analysis	Population: patients and physicians			Adoption of core MU elements can reduce ADEs, with cost savings that recoup 22% of IT costs
			Perspective: not stated			

### Description of studies

Eight studies published between 1975 and 2013 were identified for inclusion in this review. The studies retrieved were conducted in the USA (6), the UK (1), and Belgium (1). All studies were conducted in a secondary care (hospital) setting; however, the studies were set in different hospital departments including the Emergency Department, Surgery, and Medicine. Six of the included studies focused their analyses on computerised data collection systems, and the remaining two focused on information usage in order to create performance indicators.

Four studies focused on the costs and benefits of data collection using information communication technology (ICT) compared to normal practice (hand-written patient records) [6-9]. Two studies examined how already implemented ICT systems can be improved to collect better quality data [9,10], and the two remaining studies [11,12] investigated how information collected by ICT can be analysed for further performance monitoring purposes.

### Costs

The included studies reported costs in various ways. Two studies [6,9] calculated the costs based on the differences between the computerised system and the manual system. Two studies [11,12] reported the differences in the total costs of a hospital-acquired medical event before and after the adoption of 1) basic electronic medical records (2011 paper) and 2) a quality indicator (2013 paper). The study by Klimt *et al*. [7] estimated the costs of transcribing by taking into account the average length of a patient record, true transcriber costs (salary, benefits), and productivity. Tierney *et al*. [8] reported total costs including bed costs, test costs, drug costs, and other costs; however, no more information about the types of costs included in ‘other’ were reported in the published study. Willems *et al*. [10] measured the financial impact of a specifically designed follow-up programme for prophylactic antibiotic use by analysing the costs of drug usage before and after implementation. Barnes *et al*. [13] reported changes in the average subsequent hospital care revenue per trauma service admission rather than focusing on costs. Of the eight included studies identified, five reported start-up costs or associated IT costs in their analysis [7-9,11,12]. However, it must be noted that technology has advanced rapidly since the mid-1980s and has become relatively less expensive over time. For example, the first computers implemented in business were very expensive and could cost thousands, however today’s computers, which are more powerful and efficient, are installed for as little as a few hundred pounds. Therefore, it is not possible to compare the costs of data collected by systems that were made more than 30 years ago with systems produced only a few years ago.

### Effects of interventions

The intervention of interest is data collection for performance monitoring purposes and the effects this intervention may have in terms of cost benefits, service delivery benefits, and patient benefits.

All eight studies included in this review report or, at least, discuss the benefits of improved data collection in hospitals. Six studies [7,8,10-13] discuss the potential benefits that may be gained by both the hospital and the patient and the remaining two papers [6,9] report the benefits generated to the hospital only.

Overall, the studies show that it is difficult to measure the benefits of improved data collection systems. Three studies measured benefits by using hospital length of stay. Tierney *et al*. [8] found that hospital length of stay was reduced by 0.89 days when the computerised system was implemented. Barnes *et al*. [13] also measured hospital length of stay but found no difference. Encinosa and Bae [12] measured patient benefits in terms of reduced 30-day stay for heart attack, heart failure, and pneumonia. Encinosa and Bae [12] measured the benefits by quantifying the amount of averted adverse drug events occurring in hospital. Three studies [7,10,13] infer the potential benefits to the patient from the results. For example, Barnes *et al*. [13] assumed that improved documentation results in higher quality-of-care, and Willems *et al*. [10] stated that, due to the intervention, ‘more appropriate drug administration is likely to have a beneficial effect on antimicrobial resistance, rates of adverse drug events, length of stay in hospital and mortality rates’. One paper by Philp *et al*. [9] states that ‘patient benefits can only be inferred, but not proven’ and do not make any speculations as to what these might include.

### Quality assessment

Overall, the reporting quality of the studies included in this review is mixed. All studies clearly reported the intervention that was being evaluated and what it was being compared against. The objectives of each study were also clear and easy for the reader to follow. The setting and target population were also stated transparently. However, other areas were reported with varying degrees of success.

The costs reported by Klimt *et al*. [7] were detailed, reporting those associated with the day-to-day running of the Dictaphone-transcribing technology and its set-up. Tierney *et al*. [8] also reported start-up costs of the microcomputer workstation intervention, but this was stated as an approximation and lacked sufficient detail. Four studies [6,9,11,12] were not transparent in their reporting of what costs where included in their analyses. For example, Encinosa and Bae [12] reported that they include ‘all hospital costs except physician and laboratory costs’ [12]; however, they are not explicit in stating what costs have been included and also they do not provide any justifications as to why they did not include physician and laboratory costs.

The measurements of benefits vary throughout the studies included in the review. Four studies [8,11-13] attempted to measure the benefits through more commonly used units such as hospital length of stay and the probability of death or readmission. On the whole, these studies clearly stated the outcome measures of interest; however justifications and explanations concerning the reasons behind their chosen measurement unit are not reported clearly. Holloway *et al*. [6] gave a detailed description of why they chose to measure effectiveness on the basis of completeness, timeliness, and operability. In contrast, Klimt *et al*. [7], who also commented on the effectiveness of the Dictaphone system through benefits such as completeness, timeliness, and operability, did not provide reasons for their choice of effectiveness measurements. Philp *et al*. [9] focused on the quantification of benefits to the hospital by eliciting nurses’ preferences. Finally, one paper by Willems *et al*. [10] did not provide a measure of effectiveness and instead stated that the benefits associated with data collection can only be speculated from their results.

The results reported by the studies are clear, but they are limited in their generalisability. Whilst, overall, the results from all of the studies, largely, present a positive effect from the installation of improved data collection systems, the lack of agreement regarding how to measure benefits best and what costs should be included makes it difficult to draw any firm conclusions.

## Discussion

### Main findings

The feasibility of collecting the relevant MDS elements is always a limitation of performance monitoring using KPIs. This systematic review aimed to synthesise the evidence base concerning the costs of hospital data collection for performance monitoring using KPIs and to identify hospital data collection systems that have proven to be cost-minimising. The main finding of this review is that the evidence base is limited regarding the impact of data capture for performance monitoring purposes and how the data is collected, recorded, and used in a hospital setting. Overall, the studies identified and included in this review weakly indicate that the collection of hospital administrative data and improvements in recording data (installation of computerised systems) can be cost-saving and potentially provide benefits to both hospital management and patients [7-13]. The review also summarises how economic evaluations of data collection systems measure the associated economic costs and benefits. The methods utilised are inconsistent throughout the studies included in this review but that may be due to the lack of research completed in this area.

Unsurprisingly, the outcomes of the review also suggest that the costs and benefits associated with data collection are largely driven by the advance of ICT. The progression of technology, to some extent, is reflected by the year in which the study was performed. The four earliest included studies focused on the costs and benefits of data collection using ICT compared to normal practice (hand-written patient records) [6-9]. Two, more recent, studies discuss how already implemented ICT systems can be improved to collect better quality data [10,13]. The remaining two included studies discussed how the information collected by ICT can be analysed for further performance monitoring purposes [11,12]. A surprising finding of the review is that there seems to be a distinct lack of studies that evaluate ICT data collection systems.

It is worth stating that more general studies have focussed on the costs (and benefits) of data collection processes, for example, Hillestad *et al*. [14], Kaushal *et al*. [15], and Himmelstein *et al*. [16]^a^.

On a final note, it is interesting that Tierney *et al*. [8] and Philp *et al*. [9] draw attention to staff attitudes in regards to the implementation of new data collection systems. Tierney *et al*. [8] state that ‘systems can only affect costs and quality of care if physicians use them, which will only happen if “costs” are minimised and offset by perceived benefits’. It is, therefore, an imperative that studies take into account the usage of these systems by physicians and hospital staff since that alone may determine the cost-effectiveness of such a system.

### Limitations

Firstly, there is currently no reference standard when reporting the costs associated with each intervention. Some of the included studies in this review [8,9] fail to clearly report the individual costs included in their analysis making it difficult for the reviewer to fully understand how the costs were calculated. Other included studies are not transparent when reporting the setup costs associated with an intervention such as the installation and maintenance of IT systems [6,8,10] bringing into question the validity of the costing results. Another important issue concerns the potential for publication bias since almost all hospitals must be engaged in this activity and have some estimate of the cost of their data collection.

It is also important to mention that none of the studies that analysed computerised technology extrapolated the cost results over time to take into account the depreciation of technology, future maintenance costs, or the cost of upgrading the system.

Secondly, the studies highlight the difficulty in measuring the level of quality of care associated with data collection and, as a result, the studies are not consistent in their reporting of this. As mentioned above, one study measures benefits using averted adverse drug events, and three studies measure quality of care using hospital length of stay with some success, but the remaining studies admit that they can only speculate the potential expected benefits of improved quality of care via data collection [9]^.^. As a result, four studies [9-12] acknowledge and recommend that more research is completed to explore the impact of data collection on quality of care.

## Conclusions

Given the limitations of this systematic review, it is difficult to conclude whether improvements in data collection systems can save money, increase quality of care, and assist performance monitoring of hospitals. Nevertheless, the results are positive and hint that data collection improvements may lead to cost savings and derive benefits for both the hospital and patient. The review has also highlighted that there is no standard reference of how to measure the benefits and costs associated with data collection; however, it is suggested that studies work towards being more transparent when reporting the methods used and the results obtained. Overall, there is a need for more research regarding the costs and benefits associated with the installation or improvement of data collection systems for performance monitoring purposes.

## Endnote

^a^We thank an anonymous referee for this observation.

## Appendix

1. Physicians
2. Nurses
3. Medical Staff, hospital
4. Exp Hospital
5. Exp Hospital Department
6. Or/1-5
7. Hospital, animal
8. 6 not 7
9. Exp Data Collection
10. Exp Medical Records Systems, Computerised
11. Exp Quality Indicators, Healthcare
12. Public Health Administration
13. Or/9-12
14. 8 and 13
15. Economics/
16. exp ‘costs and cost analysis’/
17. Economics, Dental/
18. exp economics, hospital/
19. Economics, Medical/
20. Economics, Nursing/
21. Economics, Pharmaceutical/
22. (economic$ or cost or costs or costly or costing or price or prices or pricing or pharmacoeconomic$).ti,ab.
23. (expenditure$ not energy).ti,ab.
24. value for money.ti,ab.
25. budget$.ti,ab.
26. or/15-25
27. ((energy or oxygen) adj cost).ti,ab.
28. (metabolic adj cost).ti,ab.
29. ((energy or oxygen) adj expenditure).ti,ab.
30. or/27-29
31. 26 not 30
32. letter.pt.
33. editorial.pt.
34. historical article.pt.
35. or/32-34
36. 31 not 35
37. exp animals/ not humans/
38. 36 not 37
39. bmj.jn.
40. ‘cochrane database of systematic reviews’.jn.
41. health technology assessment winchester england.jn.
42. journal of medical economics.jn.
43. or/39-42
44. 38 not 43

## Abbreviations

CHEERS: Consolidated Health Economic Evaluation Reporting Standards
KPI: key performance indicators
MDS: minimum dataset
QA: quality assessment
